# Bacterial diversity of the rock-water interface in an East Antarctic freshwater ecosystem, Lake Tawani(P)^†^

**DOI:** 10.1186/2046-9063-9-4

**Published:** 2013-02-01

**Authors:** Jonathan P Huang, Ashit K Swain, Robert W Thacker, Rasik Ravindra, Dale T Andersen, Asim K Bej

**Affiliations:** 1 Department of Biology, University of Alabama at Birmingham, 1300 University Blvd., CH464, Birmingham, AL 35294-1170, USA; 2 Geological Survey of India, Antarctic Division, NH-5P, NIT, Faridabad 121001, India; 3 National Centre for Antarctic & Ocean Research, Head Land Sada, Vasco-da-Gama, Goa, India; 4 Carl Sagan Center for the Study of Life in the Universe, Mountain View, CA 94043, USA; 5 Current address: Panikkar Professor and Chairman, INSA-SCAR National Committee, Earth System Science Organization (MoES)Lodi Road, New Delhi 110 003, India

**Keywords:** Culture-independent, Culture-dependent, Antarctic freshwater lake, 16S rRNA, bTEFAP, Pyrosequencing, Metagenomics

## Abstract

Schirmacher Oasis is one of the few ice-free plateaus in East Antarctica that maintains a unique distribution of over 120 microbial-rich, dynamic freshwater lakes, most of which are unexplored. In this study, we describe the bacterial diversity of the rock-water interface in Lake Tawani(P) using culture-independent Bacterial Tag Encoded FLX Amplicon Pyrosequencing (bTEFAP), clone library construction, and culture-based analysis targeting the eubacterial 16S rRNA gene. Lake Tawani(P)was formed in a fossil valley by the accumulation of snow and glacial melt through surface channels into a low-catchment depression. Overall this lake exhibited thirteen bacterial phyla and one-hundred and twelve genera. The Qiime bioinformatics analysis on the bTEFAP alone exhibited higher coverage of the bacterial composition in Lake Tawani(P) than the clone library construction or culture-based methodology. Particularly due to the higher sensitivity of the bTEFAP approach, we detected and differentiated members of the phyla: Chloroflexi, Gemmatimonadetes, Planctomycetes, Nitrospira, and Candidate Division TM7 that other methods were unable to reveal. Nevertheless we found that the use of multiple approaches identified a more complete bacterial community than by using any single approach. Investigating the bacterial diversity of the Schirmacher Oasis lakes, especially those connected through surface channels and encompassed by valleys, will help unravel the dynamic nature of these unique seasonal, freshwater lakes, which potentially harbors highly adapted bacterial taxa with defined ecological functions.

## Introduction

Antarctic oases are rocky landscapes devoid of perpetual ice-cover and typically surrounded by glaciers, nunataks and mountains. A wide variety of freshwater lakes are found in Antarctic oases, the surface ice of some of them completely melts during the Austral summer months, while others remain permanently ice-covered [1,2]. A diverse group of heterotrophic bacterial communities, cyanobacterial mats and lichens inhabiting the ice, waters, sediments, endoliths and soils of these Antarctic oases provide valuable information about the structure and function of the microbial ecosystems in the Antarctic extreme conditions [2-7]. The Schirmacher Oasis (latitude 70°43’50˝ S to 70°46’40˝ S and longitude 11°22’40˝ E to 11°54’25˝ E) is approximately 17 km long and 2–3 km wide exposed bedrock spanning East to West of the Central Dronning Maud Land of East Antarctica and consists of over 120 freshwater lakes [8]. The Oasis is surrounded by the Schirmacher Hills, a continental ice-sheet in the south and an ice-shelf on the north [8,9]. The average annual temperature in this Oasis has been reported at −11°C ± 1°C and the mean wind velocity is ~10 m/s [10]. Most lakes in the Schirmacher Oasis are landlocked freshwater category with varying depths that were formed by natural processes of ice erosion and melt water from snow beds and ice slopes [2,8,9]. Although the general impression of these freshwater lakes in Schirmacher Oasis is that they are relatively young, the ^14^C isotopic analyses suggest that these lakes emerged approximately 10,000 years before present (BP) during the global warming event of the Holocene epoch [1,11,12]. As early as 1966, Komarek and Ruzicka [13] reported that the ice-covered lakes (0°C to −5°C) in Schirmacher Oasis are highly productive with an abundance of blue-green algae. Subsequent studies showed that many of these lakes harbor cyanobacteria, fresh water algae and heterotrophic bacteria [10,13-23]. Unlike the perennially ice-covered lakes in the Antarctic continent, the structure and function of the Schirmacher Oasis freshwater lakes are modulated by wind, seasonal freeze-thaw cycles, and intermixing of lake waters with snow and ice melt through channels that connect these lakes. Therefore, the community dynamics of the bacterial ecosystems in the Schirmacher Oasis lakes are most likely driven by the interaction between the annual weather cycles and the microorganisms inhabiting these lakes.

Recently the application of next-generation high throughput sequencing (hereafter NextGen HTS) has been applied to study environmental microbial communities [24-26]. However, limited studies have used this technology to investigate the Antarctic microbial ecosystem [26,27]. Furthermore there has been no study that utilized this technology to investigate the microbial diversity in Antarctic freshwater seasonal lakes as most studies have focused on traditional culture-independent clone library construction or molecular-based culture-dependent approaches. Other studies have investigated the advantages and disadvantages of taxonomic identification of microbes using culture-based approach [28,29], culture-independent clone libraries [24] and NextGen HTS approach [24,30,31] in heterologous ecosystems, but to the best of our knowledge no study has combined all three approaches on a single microbial community. We predict that applying all three methods in a single ecosystem [Lake Tawani (Projected) - described hereafter Lake Tawani(P)] will allow us to determine how the different methodologies compare in elucidating the bacterial community with adequate coverage. In this study, we describe the bacterial diversity in a recently-formed (personal communication Indian Geological Survey and Indian Antarctic Maitri Station) freshwater ecosystem, Lake Tawani(P), using culture-independent 16S rRNA Bacterial Tag Encoded FLX Amplicon Pyrosequencing (bTEFAP) on the metacommunity DNA. In addition, we have compared a 16S rRNA-based clone library and cultured colonies with the bTEFAP approach. Elucidating the bacterial communities in Lake Tawani(P) is an essential first step in predicting the putative functions and significance in this seasonally ice-covered lake ecosystem and among similar lakes particularly those connected through surface channels in this unique oasis and other locations in the Antarctic continent.

## Materials and methods

### Study site and sampling

**Figure 1:**
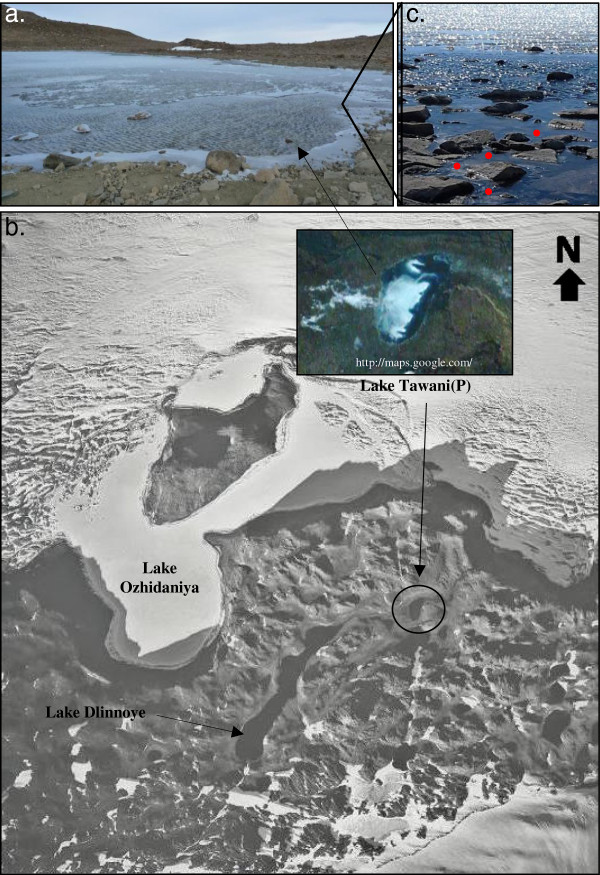
**a: Partial picture of Lake Tawani(P) in Schirmacher Oasis, Antarctica.b**: The satellite picture of Lake Tawani(P) and its vicinity. Note the continuation of the channel from the Lake Tawani(P) to the ice-shelf along a lineament marking the presence of a fossil valley and beyond connecting landlocked and proglacial lakes. Lake Tawani(P) is situated in this fossil valley and acts as a catchment for the overflow of snow and glacial melt feeding the lake. The inset is the Google Earth ( http://maps.google.com) satellite picture showing Lake Tawani(P). **c**: Actual location where samples were collected in Lake Tawani(P). The red dots indicate the rock-water interface in which sample collection occurred.

Lake Tawani(P) (70°45’ 13.6″ S; 11°41’ 25.7″ E; area 0.4 km^2^; 4 meters depth) is located 1.3 miles N/NW from the permanent Indian Station *Maitri* (70°46’32″S; 11°43’52.10″E), 0.6 miles W/SW from a freshwater Lake Dlinnoye (70°45’16″S; 11°39’52″E); 0.5 miles E/NE of Schirmacher glacier (70°45’05″S; 11°42’40″E), and 0.6 miles south of Lake Ozhidaniya (70°44’41″S; 11°41’19″E) in the Schirmacher Oasis of the East Antarctic Dronning Maud Land (Figure 1). The water mixed with the suspended sediment samples at a 10–15 cm depth near the rock-water interface where freeze-thaw cycles occurs through diurnal temperature variation (Figure 1) were collected aseptically in sterile Whirl-Pak™ bags and Nalgene™ bottles in November 2008. The temperature and other physical characteristics of the water samples were recorded at the time of collection: 0.4°C±0.02°C, pH 9.1±0.15, and refractive index <1.333. The samples were kept cold at 2–4°C in an insulated bag with blue-ice packs during collection, and then transferred at −20°C to the *Maitri* Station; samples were transported and stored at that temperature in the lab at UAB until used. Triplicate samples were collected, pooled and used for all experiments.

### Bacterial enumeration by direct plate count

Water samples (100 μl) from Lake Tawani(P) were spread plated onto ten pre-chilled R2A agar plates (Difco/Becton Dickinson) and incubated at 10°C for 10–15 days and the total number of colonies were counted. Acridine orange direct microscopic counts were conducted under a Lietz™ Diaplan epifluorescent microscope using the procedure described by Daley and Hobbie [32].

### Purification of DNA

For culture-independent metacommunity DNA analysis, Lake Tawani(P) water samples (10 ml) were centrifuged at 10,000 *g* for 10 min at 4°C to pellet bacterial cells resuspended in sterile distilled water, boiled for 10 min to release DNA and then quickly transferred on ice. Metacommunity DNA was then subjected to downstream genomic analyses.

### Parallel bacterial tag-encoded FLX-Amplicon pyrosequencing (bTEFAP)

Bacterial tag-encoded FLX amplicon pyrosequencing (bTEFAP) was performed by Research and Testing Laboratories (RTL, Lubbock, TX) ( http://www.researchandtesting.com). Oligonucleotide primers 341F (5’–CCT ACG GGA GGC AGC AG–3’ [33] and 907R (5’–CCG TCA ATT CMT TTG AGT TT–3’ [34] that targets the V3-V5 region of the 16S rRNA [35] were used for pyrosequencing. The initial generation of the sequencing library was performed utilizing a one-step PCR with a total of 30 cycles, a mixture of Hot Start and HotStar high-fidelity *Taq* DNA Polymerases, and amplicons originating and extending from the 341F oligonucleotide primer. Then tag-encoded FLX amplicon pyrosequencing were conducted on a Roche 454 FLX with Titanium reagents (Roche, Indianapolis, IN) following the titanium protocol described by the manufacturer.

### Sequence processing and bioinformatics

The pyrosequence reads were analyzed and processed using QIIME 1.3.0 workflow ( http://www.qiime.org/) as described by Caporaso et al. [36]. Briefly, QIIME processes 16S rRNA gene sequences, clusters them using uclust at 97% sequence similarity ( http://drive5.com/usearch/usearch3.0.html), classifies using the Ribosomal Database Project (RDP) classifier at >50% confidence ( http://rdp.cme.msu.edu/), aligns them using Pynast ( http://qiime.org/pynast), constructs phylogenetic trees using FastTree2 ( http://www.microbesonline.org/fasttree/), and generates data summaries of the proportions of taxa.

### PCR amplification and Clone libraries of the 16S rRNA using Sanger sequencing

An aliquot (5 μl) of the originally centrifuged and boiled metagenomic DNA extract served as a template for the PCR reaction targeting the eubacterial 16S rRNA gene using the universal forward oligonucleotide primer 18F (5’-AGAGTTTGATCATGGCTCAG-3’) and reverse primer 1509R (5’-GGTTACCTTGTTACGACTT-3’) [37]. The PCR cycling parameters were modified from Martinez et al., [37] and consisted of an initial denaturation step at 95°C for 5 minutes followed by 25 cycles of denaturation at 95°C for 1 min, primer annealing at 55°C for 1 min and primer extension at 72°C for 2 min. Following 25 cycles of amplification, a final extension step was held at 72°C for 10 minutes. The amplified DNA samples were stored at 4°C until used for subsequent experiments. To avoid sample contamination, proper precautions including the standard microbiological aseptic protocols were followed. DNA extraction and amplification were conducted under an UV-sterilizing laminar flow cabinet. For every PCR amplification series, one reaction, which consisted of all the necessary PCR reagents except for a DNA template, served as a negative control.

The amplified PCR products were ligated overnight onto the pGEM-T Easy™ vector and transformed into *Escherichia coli* JM109 (ATCC 53323) competent cells following the protocol described by the manufacturer (Promega). The putative transformed white colonies were plated on Luria-Bertani (LB) agar plates supplemented with ampicillin (50 μg/ml), 100 μl of IPTG (100 mM) and 20 μl of X-gal (40 mg/ml) then picked and resuspended and boiled for 10 min to release plasmid DNA, which was then subjected to PCR amplification using the 16S rRNA oligonucleotide primers to confirm the correct size DNA inserts. Two hundred and thirty-two colonies with correct size DNA inserts were purified using Genscript Quickclean 5 M™ Miniprep Kit (Genscript) and sequenced using M13 forward and reverse primers and standard capillary sequencing methods at the UAB Center for AIDS Research (UAB CFAR; http://www.uab.edu/cfar/dna-sequencing-core).

### Culture-based approach

For culture-based analysis, samples (100 μl each) were spread-plated on R2A agar plates (BBL), incubated at 15°C for 10 days for colonies to appear. Individual bacterial colonies from agar plates were randomly selected (*n*=270), each resuspended in 25 μl sterile distilled water and boiled for 10 min to release DNA, and then quickly transferred on ice. An aliquot (3 μL) of the boiled sample was then PCR-amplified which targeted the 16S rRNA using the oligonucleotide primers and PCR parameters described above.

### Chimera checking and accession numbers based on Sanger 16S rRNA gene sequences

Sequences were compared to known sequences using the NCBI database Basic Local Alignment Search Tool (BLAST) [38]. Additional comparison was done with the RDP ( http://rdp.cme.msu.edu/). In order to determine if chimeras were present in these constructs, all sequences were subjected to the Bellerophon chimera detection program [39] and Chimera Slayer [40]. Any possible chimeras detected were removed from the constructs. After removal of chimera, the 16S rRNA gene sequences were submitted to GenBank under the accession numbers [GenBank:JF714490-JF714543] and [GenBank:JF778652-JF778667].

### Statistical analysis

Operational taxonomical unit (OTU) classified at 97% sequence similarity were clustered (uclust) and the diversity (Shannon diversity index and Simpsons diversity index), evenness (Shannon evenness index – higher values reflect less variation between numbers of associated species), and richness were also determined. The Shannon diversity index [41] was defined as $H^{’}=\sum_{i=1}^{s}\left(p_{i}\text{ln}p_{i}\right)$ where *s* is the number of OTUs in the sample and *p*_*i*_ is the proportion of the organisms in the sample represented by the *i*th OTU. The Simpsons diversity index [42] was defined as *D* = 1/∑*p*_*i*_^2^ where pi is the proportion of the sample that OTU i constitutes. The Shannon evenness index was calculated as *E*_*H*_ = *H*’/*H*’_max_ = *H*’/ln *S* where *S* is the number of OTU in the sample, *H´ * is the Shannon Diversity Index, and *H*´ _max_ is the maximum value of *H*´. Chao1 estimates targeting the 16S rRNA sequences obtained from bTEFAP and Sanger sequences were calculated using Qiime 1.3.0 and EstimateS ( http://viceroy.eeb.uconn.edu/EstimateS/) respectively.

## Results

### Bacterial diversity of Lake Tawani(P) based on bTEFAP

**Figure 2:**
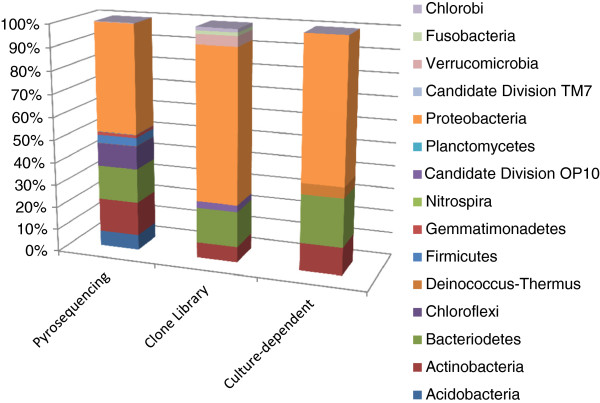
**Relative abundances of the different bacterial phyla identified in Lake Tawani(P) through culture-dependent and culture-independent (pyrosequencing and clone library) methodologies targetting the 16S rRNA gene.** Bacterial sequences were classified (>50% confidence) based on the RDP Classifier through Qiime 1.3.0 ( http://www.qiime.org/).

**Figure 3:**
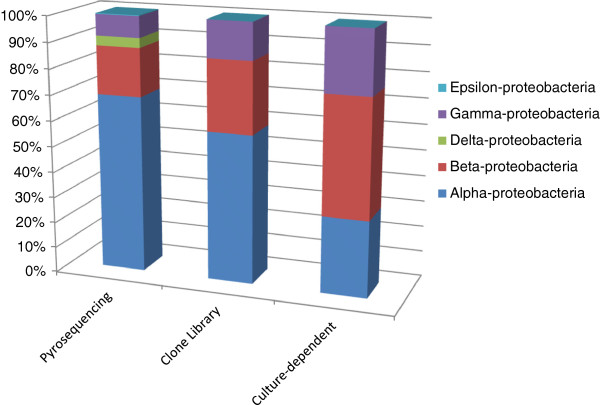
**Relative abundance of the class distribution within the phylum Proteobacteria in the Lake Tawani(P).** The stacked columns provide a comparison of the different bacterial classes identify by the culture-independent and culture-dependent methodologies. RDP Classifier determined the bacterial taxonomy with >50% confidence through Qiime 1.3.0 ( http://www.qiime.org/).

**Figure 4:**
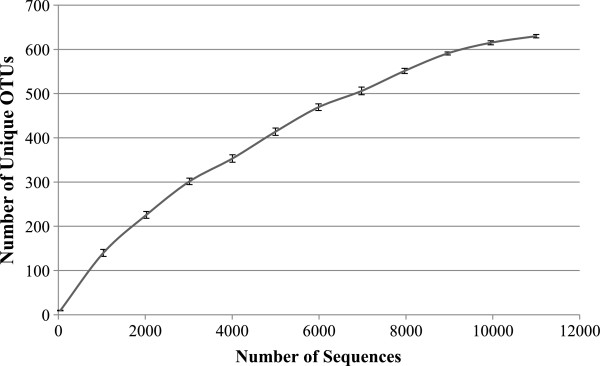
**Rarefaction curve of the partial sequences (*n*=11,235) from the bacterial 16S rRNA genes generated by pyrosequencing identified in Lake Tawani(P).** Rarefaction curves and unique OTUs (operation taxonomical units) at 3% sequence variations and standard deviations were calculated by Qiime 1.3.0.

A total of 11,235 high-quality sequences were obtained from the water samples collected from Lake Tawani(P). These sequences were clustered into 507 unique OTUs at 97% sequence similarity. In this study, the use of the 16S rRNA gene analyses resulted in the identification of 12 different phyla and 110 different genera including organisms from the Candidate Division OP10 and Candidate Division TM7 groups (Additional file 1: Table S1; Figure 2). The majority of bacterial sequences obtained from bTEFAP belonged to the phylum Proteobacteria, which represented 47.62% of the total sequences. The members of the phylum Proteobacteria fell within 4 different classes: Alpha-proteobacteria (71.52% of all microbes in the phylum Proteobacteria), Beta-proteobacteria (18.78%), Delta-proteobacteria (2.03%), and Gamma-proteobacteria (7.67%) (Figure 3). Within the class Alphaproteobacteria, the bacterial genera *Rhodobacter* and *Sphingomonas* were predominant. Following the phylum Proteobacteria, the next common groups of phyla were Bacteroidetes (15.10%), Actinobacteria (14.83%), Chloroflexi (10.21%), Acidobacteria (6.71%), Firmicutes (3.64%), Gemmatimonadetes (1.26%), and Verrucomicrobia (0.21%), while Deinococcus-Thermus, Nitrospira, Candidate Division OP10, Planctomycetes, Candidate Division TM7, and Fusobacteria all represented 0.07% of total bacterial sequences. Rarefaction curve that are rapidly approaching saturation was constructed at 3% sequence variation using Qiime 1.3.0 (Figure 4).

### Bacterial diversity of Lake Tawani(P) based on Sanger method of 16S rRNA gene sequences

The nucleotide sequence of the cloned fragments (*n=*232) of the 16S rRNA gene from culture-independent metacommunity DNA exhibited 7 phyla (Proteobacteria, Actinobacteria, Bacteriodetes, Fusobacteria, Verrucomicrobia, Chlorobi, and Candidate Division OP10) and 16 different genera (Additional file 1: Table S1; Figure 2). Similar to the pyrosequencing data, the largest number of clones belonged to the Phylum Proteobacteria at 67.32% of the total clone library. These Proteobacteria fell into 3 different classes: Alpha-proteobacteria (58.67%) including *Sphingomonas Caulobacter*, and *Brevundimonas*; Beta-proteobacteria (28%) including *Janthinobacterium*, *Duganella*, *Polaromonas*, *Variovorax*, and *Rhodoferax*; Gamma-proteobacteria (13.33%) including *Pseudomonas* and *Acinetobacter*. The next most common phylum was Bacteriodetes (15.61%), which included *Flavobacterium*, *Pedobacter*, *Prevotella*, *Hymenobacter*, and *Arcicella* (Additional file 1: Table S1). The clone frequency of the phylum Actinobacteria accounted for 6.83% of the total bacteria, followed by the phylum Verrucomicrobia (4.39%) and finally the phyla Fusobacteria, and Chlorobi each represented approximately 1.46% of the total bacterial community. The 16S rRNA gene sequences amplified from cultured isolates (*n=*247) revealed 4 phyla (Proteobacteria, Actinobacteria, Bacteriodetes, and Deinococcus-Thermus) and 15 different genera (Additional file 1: Table S1).

### Bacterial enumeration and statistical analysis

The viable plate count of the Lake Tawani(P) samples grown at 10°C on R2A agar plates exhibited an average of 2 x 10^3^ ± 1.5 cfu/ml. Acridine orange direct count (AODC) exhibited a bacterial count of 5.4 x 10^3^ cells per ml. Both Shannon and Weaver, and Simpsons diversity indices using the 16S rRNA culture-independent analyses of community DNA exhibited a more diverse eubacterial community compared to the culture-based approach (Table 1).

**Table 1:** Shannon diversity, Simpsons diversity, Shannon Evenness, OTU richness and Chao1 Estimate calculated based upon the eubacterial 16S rRNA gene sequences of rock-water interface samples from Lake Tawani(P) using culture-dependent and culture-independent methods

	**Shannon diversity index**	**Simpsons diversity index**	**Shannon Evenness index**	**OTU richness**	**Chao1 estimate**
16S rRNA culture-dependent	2.58	0.92	0.95	16	15
16S rRNA clone library	2.09	0.76	0.72	64	22
16S rRNA pyrosequencing	7.20	0.99	0.82	497	961

## Discussion

Spatial and temporal variation in snow and ice cover across Antarctica markedly affects all ecological variables, including the composition of bacterial assemblages and their ecosystem functions [43]. The ice-free Antarctic oases often represent Earth’s past geologic and climatic evolution thus are good ecological indicators for future global climate change [12,44-49]. Since 1961, the mean annual temperature increased by 1°C from −11°C to approximately −10°C ( http://south.aari.nw.ru/data/data.asp?lang=0&station=1) in the Schirmacher Oasis may have resulted in an increased melting of snow, glacial, and continental ice thereby affecting the distribution and organization of the lacustrine systems. We predict that Lake Tawani(P), which is situated in a fossil valley (Figure 1), initially existed as a low-catchment depression, progressively filled with water from increased glacial ice and snow melt through visible surface channels and eventually became a permanent landlocked freshwater lake. In this study we have determined the 16S rRNA gene sequence-based bacterial diversity using culture-independent (bTEFAP and clone library construction) and culture-dependent methodologies on rock-water interface samples from Lake Tawani(P). In addition we have compared the resulting bacterial communities from Lake Tawani(P) with previously reported bacteriological profiles from sediment samples in the nearby lake L6 in Schirmacher Oasis and water samples from two distantly located Lundström Lake and Forlidas Pond in the Transantarctic Mountains.

The use of the bTEFAP approach targeting the V3-V5 regions of the 16S rRNA revealed that the majority of eubacteria belonged to the phylum Proteocbacteria, followed by Bacteriodetes, and Actinobacteria (Figure 2). Within the phylum Proteobacteria, the class Alpha-proteobacteria dominated for both bTEFAP (72%) and the clone library (58.67%) while the culture-based approach revealed a relative higher distribution of members from the class Beta-proteobacteria (45.83%) (Figure 3). Newton et al. [50] suggested that generally most members of the phyla Proteobacteria (Alpha and Beta-proteobacteria), Bacteriodetes, and Actinobacteria are restricted to only freshwater ecosystems and thus represent native organisms. Moreover the bacterial composition fits with the hypothesis that Lake Tawani(P) formed as a recent increase in temperature caused by an accrual of seasonal glacial and snow melt, which in turn resulted in new lakes that contain mostly natives and not vagabonds (microbes that have a wider distribution than specifically freshwater environments; [50]) to the freshwater ecosystem.

Furthermore bTEFAP distinguished a significantly higher coverage of bacterial taxa than the clone library and culture-dependent methodologies (Additional file 1: Table S1 and Table 1). Predominant sequences identified through bTEFAP include the genera *Rhodobacter*, *Illumatobacter*, *Roseomonas*, *Haliscomenobacter*, and *Sphaerobacter*. All of which were not identified by the clone library construction or culture-based approach. Additionally 5 phyla and 89 other genera were not differentiated by clone-library construction or culture-based methodologies (Additional file 1: Table S1). Moreover 439 unique OTUs (87% of total OTUs) were only identified by bTEFAP, 48 OTUs were identified in both the clone library construction and bTEFAP, and 9 distinct OTUs were detected in all three methodologies. Although 8 OTUs were identified by either clone library or culture-dependent approaches but not through bTEFAP (Additional file 2: Table S2). This supports the notion that the high sequence coverage by NextGen HTS allows a more complete cataloging of the bacterial community as compared to clone library and culture-dependent methodologies [24,31]. While other common genera such as *Sphingomonas*, *Polaromonas*, and *Arcicella* (OTU# 20, 125, 13 respectively; Additional file 2: Table S2) found through bTEFAP (within top 20 most abundant genera), they were also identified by clone library construction but not by the culture-based approach. Additionally, the genera *Caulobacter*, *Actinomyces*, *Fusobacteria*, and *Verrucomicrobia* (OTU # 443, 324, 314, and 283; Additional file 2: Table S2) were not identified by the culture-based approach, but were identified in both the clone-library construction and bTEFAP. Although there were some differences between the clone library construction and culture-based approaches, it is notable that unlike other studies [51], Lake Tawani(P) exhibited a significant number of bacterial genera that were found to be similar between the two methodologies. Interestingly, the genera *Propionibacterium* (OTU# 26), *Acinetobacter* (OTU# 222) and *Chlorobi* (OTU# 392) were only found in the clone-library and not through the other approaches while *Deinococcus* (OTU# 363) was present in pyrosequencing and culture-based but not through the clone library construction. The use of culture-dependent in this study was conducted solely on R2A agar medium and colonies were collected at one time point which may have limited the growth of certain microorganisms and thus provide a decreased diversity count. Despite this caveat, previous studies on the bacterial diversity found in Antarctic freshwater ecosystems have specifically used R2A for isolating Antarctic bacteria [52-54]. Overall the greater sensitivity of pyrosequencing gave a higher bacterial coverage than the clone-library construction or culture-based methodology though the use of multiple approaches deciphered a more complete bacterial assemblage than by using any single approach. It is interesting to note that ubiquitous freshwater groups such as Actinobacteria-acl group [55,56] were not identified by any of the three methodologies. The geochemistry and nutritional composition of the region may be the governing factor for this observation, which requires further investigation. Interestingly, the Actinobacterial sequences have been found only in Ace Lake, Antarctica which is unusual because this lake has been reported to have originated as a salt water lake [26]. Further exploration of the Actinobacteria sequences will be necessary to investigate the ubiquitous nature of these microorganisms in extreme environments.

Analysis of the 16S rRNA gene sequences from lake L6 (study of the bacterial diversity in lake sediment; 70°45’20.25″S; 11°35’52.38″E) [10], which is also located in the Schirmacher Oasis, exhibited similar bacterial phyla to Lake Tawani(P). Both environments consisted of the phyla Proteobacteria, Bacteroidetes, Actinobacteria, and Firmicutes. Additionally, the genera *Janthinobacterium*, *Pseudomonas*, *Flavobacterium*, *Arthrobacter*, *Pedobacter*, *Polaromonas*, *Rhodopseudomonas*, and *Paenibacillus* were common between the two lakes. Despite these similarities, the phyla Fusobacteria, Verrucomicrobia, Chlorobi, Chloroflexi, Acidobacteria, Gemmatimonadetes, Deinococcs-Thermus, Nitrospira, Planctomycetes, Candidate Division OP10, and Candidate Division TM7 were identified in Lake Tawani(P) but not in lake L6 [10]. Additionally, a number of bacterial genera found in Lake Tawani particularly microbes from the class Alpha-Proteobacteria were not identified in lake L6.

Both Lake Tawani(P) and the distantly located seasonal lakes of the Transantarctic Mountains (51°16'W, 82°27'S; 29°29'W, 80°27'S) [57] consisted of members from the phyla Proteobacteria, Firmicutes, Bacteroidetes, and Actinobacteria. Similar to Lake Tawani(P), the dominant class of eubacteria found in Lundström Lake and Forlidas Pond belonged primarily to the class Alpha-Proteobacteria [57]. Furthermore bacterial genera such as *Leifsonia*, *Arthrobacter*, *Brevundimonas*, *Devosia*, *Bacillus*, *Hymenobacter*, *Variovorax*, *Ramlibacter*, *Flavobacterium*, *Paracoccus*, *Sphingopyxis*, *Herminiimonas*, *Paenibacillus*, *Rhodococcus*, *Algoriphagus*, *Pedobacter*, *Carnobacterium*, *Sphingomonas*, *Janthinobacterium*, *Spirosoma*, *Sandaracinobacter*, *Polaromonas*, *Bosea*, *Lysobacter*, and *Pseudoxanthomonas* were distributed in both Lake Tawani(P) and the seasonal lakes of the Transantarctic Mountains.

Similar bacterial diversity (*Leifsonia*, *Arthrobacter*, *Flavobacterium*, *Pedobacter*, and *Janthinobacterium*) between the lakes in the Schirmacher Oasis (Lake L6 and Lake Tawani(P)) and other freshwater lakes in Antarctica (Lundström Lake and Forlidas Pond in the Transantarctic Mountains) indicate that surface channels, and constant wind (often katabatic category) causes intermixing of microbes within the freshwater lakes in Schirmacher Oasis and among other freshwater lakes in Antarctica. In addition many taxa such as Candidate Division OP 10, Candidate Division TM7, Gemmatimonadetes, and Acidobacteria are normally unculturable have been identified in Lake Tawani(P) but not in lake L6 or the seasonal lakes in the Transantarctic Mountains. Although members in the Candidate Division OP10 are found in hydrothermal vents, volcanic islands [58,59], hypersaline lakes [60], soils [61-64], fresh- and marine-waters [65-67], they are not common in the Antarctic ecosystem [68]. Similarly the members of the Candidate Division TM7 group have been identified in mature forest soil and batch reactor sludge but not in Antarctica [69]. Other unculturable phyla such as Gemmatimonadetes and Acidobacteria which represented approximately 8% of total bTEFAP reads were also identified in this study. Members of these phyla are most often found residing in soil ecosystems [23,70,71] but very few studies have differentiated these bacteria in Antarctic freshwater lakes [72,73]. These unculuturable bacteria may have ecological roles such as nutrient cycling within Lake Tawani(P) [71,74]. These differences in bacterial diversity could be due to the differences in lake physico-chemical parameters but also due to the deep sequence coverage by the use of NextGen HTS conducted in this study.

In conclusion, microbes are the dominant organisms and play essential roles in ecosystem functioning in Antarctic freshwater lakes [26]. Due to the extreme cold, dry, high solar UV radiation and wind, Antarctic ecosystems present high selective pressure where many microorganisms cannot adapt while others flourish. Unlike the West Antarctic McMurdo Dry valley lakes, the East Antarctic lacustrine ecosystems remain largely unexplored. The Schirmacher Oasis is a unique lacustrine system with over 120 freshwater lakes where constant high wind, snow and glacial melts runoff during the Austral summer months through surface channels causes an intermixing of the lake waters and often results in appearance of new lakes and ponds [8,74]. We believe that excessive snow and glacial melt during the recent years due to the relatively higher temperature (an average increase of +1°C) led to the appearance of Lake Tawani(P), which rapidly established a diverse and sustainable bacterial ecosystem. Due to the overall commonality of microorganisms found in Lake L6 [10] and Lake Tawani(P), further study of the bacterial diversity among additional freshwater lakes in the Schirmacher Oasis, particularly those connected through surface channels, may unfold the extent of intermixing and exchange of microorganisms among lakes. This will help identify persisters that contribute to a stable functioning ecosystem in this unique East Antarctic oasis.

## Competing interests

The authors declare that they have no conflict of interest.

## Authors’ contributions

JPH conducted the experimental work/design, data analysis, and drafted the manuscript. AKS and RR, Antarctic scientists, were involved with the Antarctic sample collection, logistics and lake surveys. RWT, a molecular ecologist, was involved with data analysis. DTA, an Antarctic limnologist, was involved in the determination and interpretation of the physico-chemical characteristics of Lake Tawani(P). AKB was involved in the experimental design, data analysis, and finalized the manuscript. All the authors have read and approved the final manuscript.

## Supplementary Material

Additional file 1: Table S1 Taxonomic identification of bacteria isolated from Lake Tawani(P). Bacteria were identified using culture-dependent and culture-independent methodologies targeting eubacterial16S rRNA gene. Click here for file

Additional file 2: Table S2 Differentiation of OTUs classified at 97% sequence similarity through culture-dependent and culture-independent methodologies targeting the eubacterial 16S rRNA gene. OTUs were created with uclust through the Qiime 1.3.0 bioinformatics pipeline. Click here for file
