# Supplementary material for: Bacterial diversity of the rock-water interface in an East Antarctic freshwater ecosystem, Lake Tawani(P)†
Source: Aquat Biosyst. 2013 Feb 1;9:4. doi: 10.1186/2046-9063-9-4 (PMC3740781; doi:10.1186/2046-9063-9-4)
Supplement: Additional file 1: Table S1 — Taxonomic identification of bacteria isolated from Lake Tawani(P). Bacteria were identified using culture-dependent and culture-independent methodologies targeting eubacterial16S rRNA gene. [file 2046-9063-9-4-S1.pdf]

Tables: Huang et al., 2013. Microbial Diversity of the Rock-Water Interface in an East Antarctic Freshwater Ecosystem, Lake Tawani(P)[†].

Supplementary Table 1: Taxonomic identification of bacteria isolated from Lake Tawani(P). Bacteria were identified using culture-dependent and culture-independent methodologies targeting eubacterial 16S rRNA gene.

	16S rRNA culture-dependent (n=247)	16S rRNA clone library (n=232)	16S rRNA Pyrosequencing (n=11,235)
**Phylum Proteobacteria**	+	+	+
**Class: Alpha-proteobacteria**	+	+	+
Acidisphaera	-	-	+
Amaricoccus	-	-	+
Belnapia	-	-	+
Bosea	-	-	+
Brevundimonas	+	+	+
Catellibacterium	-	-	+
Caulobacter	-	+	+
Devosia	-	-	+
Filomicrobium	-	-	+
Geminicoccus	-	-	+
Haematobacter	-	-	+
Hoeflea	-	-	+
Hyphomicrobium	-	-	+
Mesorhizobium	-	-	+
Methylocystis	-	-	+
Methylovirgula	-	-	+
Mythlosinus	-	-	+
Novosphingobium	-	-	+
Paracraurococcus	-	-	+
Phenylobacterium	-	-	+
Porphyrobacter	-	-	+
Pseudorhodobacter	-	-	+
Rhodobacter	-	-	+
Rhodoplanes	-	-	+
Rhodopseudomonas	-	-	+
Rhodovarius	-	-	+
Roseococcus	-	-	+
Roseomonas	-	-	+
Rubellimicrobium	-	-	+
Rubritepida	-	-	+
Sandaracinobacter	-	-	+
Sandarakinorhabdus	-	-	+
Sphingomonas	+	+	+
Sphingopyxis	-	-	+
**Class: Beta-proteobacteria**	+	+	+
Acidovorax	-	-	+
Aquabacterium	-	-	+

Caenimonas	-	-	+
Collimonas	-	-	+
Duganella	+	+	+
Herminiimonas	-	-	+
Janthinobacterium	+	+	+
Methylibium	-	-	+
Paucibacter	-	-	+
Polaromonas	+	+	+
Ramlibacter	-	-	+
Rhodoferax	+	+	+
Variovorax	+	+	+
**Class: Delta-proteobacteria**	-	-	+
Kofleria	-	-	+
Nannocystis	-	-	+
Phaselicystis	-	-	+
**Class: Gamma-proteobacteria**	+	+	+
Acinetobacter	-	+	-
Aquimonas	-	-	+
Dokdonella	-	-	+
Frateuria	-	-	+
Fulvimonas	-	-	+
Legionella	-	-	+
Lysobacter	-	-	+
Pseudomonas	+	+	+
Pseudoxanthomonas	-	-	+
**Phylum Actinobacteria**	+	+	+
Actinomyces	-	+	+
Actinomycetospora	-	-	+
Arthrobacter	+	+	+
Blastococcus	-	-	+
Conexibacter	-	-	+
Fodinibacter	-	-	+
Humicoccus	-	-	+
Iamia	-	-	+
Ilumatobacter	-	-	+
Kineococcus	-	-	+
Marmoricola	-	-	+
Nocardioides	-	-	+
Pimelobacter	-	-	+
Propionibacterium	-	+	-
Pseudonocardia	-	-	+
Subtercola	+	+	+
Terracoccus	-	-	+
Thermoleophilium	-	-	+
**Phylum Bacteroidetes**	+	+	+
**Class: Bactereroidetes**	-	+	+
Prevotella	-	+	+
**Class: Flavobacteria**	+	+	+
Flavobacterium	+	+	+

Ornithobacterium	-	-	+
**Class: Sphingobacteria**	+	+	+
Adhaeribacter	-	-	+
Arcicella	-	+	+
Ferruginibacter	-	-	+
Flavisolibacter	-	-	+
Haliscomenobacter	-	-	+
Hymenobacter	+	+	+
Niabella	-	-	+
Parasegetibacter	-	-	+
Pedobacter	+	+	+
Rudanella	-	-	+
Runella	-	-	+
Salinibacter	-	-	+
Sediminibacterium	-	-	+
Spirosoma	-	-	+
Terrimonas	-	-	+
**Phylum Gemmatimonadetes**	-	-	+
Gemmatimonas	-	-	+
**Phylum Fusobacteria**	-	+	+
Fusobacteria	-	+	+
**Phylum Verrucomicrobia**	-	+	+
Opitutus	-	-	+
Verrucomicrobia	-	+	+
**Phylum Planctomycetes**	-	-	+
Singulisphaera	-	-	+
**Phylum Nitrospira**	-	-	+
Nitrospira	-	-	+
**Phylum Chlorobi**	-	+	-
Chlorobi	-	+	-
**Phylum Deinonococcus-Thermus**	+	-	+
Deinococcus	+	-	+
**Phylum Chloroflexi**	-	-	+
Caldilinea	-	-	+
Chloroflexus	-	-	+
Herpetosiphon	-	-	+
Longilinea	-	-	+
Sphaerobacter	-	-	+
**Phylum Firmicutes**	-	-	+
Paenibacillus	-	-	+
Staphylococcus	-	-	+
**Candidate Division OP 10**	-	+	+
**Candidate Division TM 7**	-	-	+
