# Supplementary material for: Bacterial diversity of the rock-water interface in an East Antarctic freshwater ecosystem, Lake Tawani(P)†
Source: Aquat Biosyst. 2013 Feb 1;9:4. doi: 10.1186/2046-9063-9-4 (PMC3740781; doi:10.1186/2046-9063-9-4)
Supplement: Additional file 2: Table S2 — Differentiation of OTUs classified at 97% sequence similarity through culture-dependent and culture-independent methodologies targeting the eubacterial 16S rRNA gene. OTUs were created with uclust through the Qiime 1.3.0 bioinformatics pipeline. [file 2046-9063-9-4-S2.pdf]

Tables: Huang et al., 2013. Microbial Diversity of the Rock-Water Interface in an East Antarctic Freshwater Ecosystem, Lake Tawani(P)[†].

Supplementary Table 2: Differentiation of OTUs classified at 97% sequence similarity through culture-dependent and culture-independent methodologies targeting the eubacterial 16S rRNA gene. OTUs were created with uclust through the Qiime 1.3.0 bioinformatics pipeline.

#OTU ID	16S rRNA culture-dependent (n=247)	16S rRNA clone library (n=232)	16S rRNA Pyrosequencing (n=11,235)
0	0	0	4
1	0	0	4
2	0	0	4
3	0	0	9
4	0	0	4
5	0	0	22
6	0	0	4
7	0	0	31
8	0	3	4
9	0	0	4
10	0	3	22
11	0	0	4
12	0	3	18
13	0	3	132
14	0	3	22
15	0	0	4
16	0	0	4
17	0	0	4
18	0	0	35
19	0	0	57
20	2	6	141
21	0	0	53
22	0	0	4
23	0	0	4
24	0	0	26
25	0	3	4
26	0	3	0
27	0	3	0
28	0	0	4
29	0	0	4

30	0	0	4
31	0	0	4
32	0	0	4
33	2	3	13
34	0	0	18
35	0	0	4
36	0	0	4
37	0	0	4
38	0	0	4
39	0	0	57
40	0	0	44
41	0	0	4
42	0	0	4
43	0	0	4
44	0	0	4
45	0	0	4
46	0	0	9
47	0	0	4
48	0	0	9
49	0	0	4
50	0	0	18
51	0	0	150
52	0	0	9
53	0	0	13
54	0	0	4
55	0	0	53
56	0	0	49
57	0	0	9
58	0	0	4
59	0	0	4
60	0	0	238
61	0	0	26
62	2	3	9
63	0	3	4
64	0	0	18
65	0	0	22
66	0	0	13
67	0	0	4

68	0	3	9
69	0	0	4
70	0	0	13
71	0	0	31
72	0	0	4
73	0	0	4
74	0	0	4
75	0	0	13
76	0	0	4
77	0	0	18
78	0	0	31
79	0	0	4
80	0	0	4
81	0	0	4
82	0	0	9
83	0	0	22
84	0	0	35
85	0	0	9
86	0	0	4
87	0	0	13
88	0	0	4
89	0	0	9
90	0	0	4
91	0	0	22
92	0	0	4
93	0	0	9
94	0	0	9
95	0	0	4
96	0	0	4
97	0	0	4
98	0	0	4
99	0	0	4
100	0	0	22
101	0	0	4
102	0	0	9
103	0	0	26
104	0	3	4
105	4	3	9

106	0	0	9
107	0	0	4
108	0	0	4
109	0	0	9
110	0	0	9
111	0	0	4
112	0	0	4
113	0	0	4
114	0	0	4
115	0	0	4
116	0	0	75
117	0	0	9
118	0	0	4
119	0	0	4
120	0	0	53
121	0	0	4
122	0	0	40
123	0	0	4
124	0	0	13
125	0	8	335
126	0	0	22
127	0	0	9
128	0	3	9
129	0	0	4
130	1	3	13
131	0	0	4
132	0	0	9
133	0	0	4
134	0	0	9
135	0	0	4
136	90	15	0
137	0	0	4
138	0	0	9
139	0	0	4
140	0	0	53
141	0	0	18
142	0	0	9
143	0	0	26

144	0	0	9
145	0	0	4
146	0	3	4
147	0	3	4
148	0	1	4
149	0	0	4
150	0	3	4
151	0	0	4
152	0	1	4
153	0	0	4
154	0	0	4
155	0	0	4
156	0	0	4
157	0	0	4
158	0	0	18
159	0	0	26
160	0	0	18
161	0	3	13
162	0	0	18
163	0	0	4
164	0	0	4
165	0	0	4
166	0	0	4
167	0	0	18
168	0	0	4
169	0	0	4
170	0	0	4
171	0	0	9
172	0	0	4
173	0	0	35
174	0	0	4
175	0	0	9
176	0	0	79
177	0	0	4
178	0	0	4
179	0	0	340
180	1	3	13
181	0	0	4

182	0	3	22
183	0	0	26
184	0	2	4
185	0	3	4
186	0	0	4
187	0	3	18
188	0	0	9
189	0	0	9
190	0	0	4
191	0	6	31
192	0	3	9
193	0	0	4
194	0	0	4
195	0	0	256
196	0	0	9
197	0	0	9
198	0	0	4
199	0	0	13
200	0	0	115
201	0	0	4
202	0	0	4
203	0	0	18
204	0	0	22
205	0	0	18
206	0	0	4
207	0	0	4
208	0	0	9
209	0	0	4
210	0	0	62
211	0	0	4
212	0	0	4
213	0	0	9
214	0	0	35
215	0	0	66
216	0	0	4
217	0	0	4
218	0	0	4
219	0	0	4

220	0	0	4
221	0	0	4
222	0	3	0
223	0	3	9
224	0	0	4
225	0	0	4
226	0	0	4
227	0	0	13
228	0	0	4
229	0	0	9
230	0	0	4
231	0	3	4
232	0	3	22
233	0	3	4
234	0	0	22
235	0	0	13
236	0	0	4
237	0	0	4
238	0	0	9
239	0	0	4
240	15	0	0
241	0	3	44
242	0	0	4
243	0	0	4
244	0	0	57
245	0	0	4
246	0	0	4
247	0	0	4
248	0	0	9
249	0	0	4
250	0	0	35
251	0	0	4
252	0	0	4
253	0	0	4
254	0	0	9
255	0	3	9
256	0	0	4
257	0	0	4

258	0	0	4
259	0	0	4
260	0	0	4
261	0	0	4
262	0	0	9
263	0	0	4
264	0	0	4
265	0	0	4
266	0	0	4
267	0	0	4
268	0	0	4
269	0	0	4
270	0	0	4
271	0	0	9
272	0	3	101
273	0	0	35
274	0	0	18
275	0	0	13
276	0	0	40
277	0	0	4
278	0	0	9
279	0	0	4
280	0	0	22
281	0	0	4
282	0	0	4
283	0	2	4
284	0	0	4
285	0	0	9
286	0	3	9
287	0	0	4
288	0	0	4
289	0	0	4
290	0	0	62
291	0	0	4
292	0	0	4
293	0	0	4
294	0	0	4
295	0	0	4

296	0	0	79
297	0	0	49
298	0	0	4
299	0	0	4
300	0	0	4
301	0	0	53
302	0	0	4
303	0	0	4
304	0	0	44
305	0	0	4
306	0	0	110
307	0	0	4
308	0	0	4
309	0	0	4
310	0	0	4
311	0	0	4
312	0	0	26
313	0	0	4
314	0	3	4
315	0	0	4
316	0	0	4
317	0	3	9
318	0	0	4
319	0	0	4
320	0	0	4
321	0	0	13
322	15	0	0
323	0	0	4
324	0	1	4
325	0	0	4
326	0	0	9
327	0	3	4
328	0	0	49
329	0	0	4
330	0	0	18
331	1	3	9
332	0	0	4
333	0	0	9

334	0	0	13
335	0	0	9
336	0	0	9
337	0	0	84
338	0	0	4
339	0	0	9
340	0	0	18
341	0	0	4
342	0	0	9
343	0	0	18
344	0	0	9
345	0	0	4
346	1	3	18
347	0	0	4
348	0	0	9
349	0	0	13
350	0	0	4
351	0	0	9
352	0	0	9
353	0	0	9
354	0	0	4
355	0	0	4
356	0	0	22
357	0	0	9
358	0	0	9
359	0	0	4
360	0	3	9
361	0	0	4
362	0	0	9
363	1	0	13
364	0	3	9
365	0	0	4
366	0	0	18
367	0	0	4
368	0	0	26
369	1	3	9
370	0	0	4
371	0	0	4

372	0	0	9
373	0	0	4
374	0	0	18
375	58	0	31
376	0	0	22
377	0	0	4
378	0	0	57
379	0	3	9
380	29	3	0
381	0	0	4
382	0	0	9
383	15	0	0
384	0	0	4
385	0	0	4
386	0	0	128
387	0	0	4
388	0	0	4
389	0	0	4
390	0	3	31
391	0	0	26
392	0	3	0
393	0	0	115
394	0	0	9
395	0	0	4
396	0	0	18
397	0	0	4
398	0	0	4
399	0	0	4
400	0	0	4
401	0	0	22
402	0	3	4
403	0	3	22
404	0	0	4
405	0	0	4
406	0	0	9
407	0	0	9
408	0	0	4
409	0	0	4

410	0	0	4
411	0	0	4
412	0	3	106
413	0	0	4
414	0	0	18
415	0	0	9
416	0	0	18
417	0	0	13
418	0	0	26
419	0	0	4
420	0	0	4
421	0	0	4
422	0	3	9
423	0	0	4
424	0	3	35
425	0	0	9
426	0	0	4
427	0	0	176
428	0	0	4
429	0	0	18
430	0	0	9
431	0	0	18
432	0	0	13
433	0	0	4
434	0	0	9
435	0	0	4
436	0	0	18
437	0	0	4
438	0	0	4
439	0	0	4
440	0	0	4
441	0	0	13
442	0	0	26
443	0	1	4
444	0	0	4
445	0	0	4
446	0	0	4
447	0	0	4

448	0	0	18
449	0	0	9
450	0	0	22
451	0	0	4
452	0	0	4
453	0	0	4
454	0	0	4
455	0	0	4
456	0	0	22
457	0	0	4
458	0	0	4
459	0	0	4
460	0	0	4
461	0	0	4
462	0	3	18
463	0	0	4
464	0	0	13
465	0	3	31
466	0	0	4
467	0	0	4
468	0	0	57
469	0	0	4
470	0	0	4
471	0	0	4
472	0	0	4
473	0	0	22
474	0	0	4
475	0	0	4
476	0	0	4
477	0	0	4
478	0	0	4
479	0	0	4
480	0	0	9
481	0	0	287
482	0	0	4
483	0	0	4
484	0	0	4
485	0	0	75

486	0	0	4
487	0	0	40
488	0	0	4
489	0	0	4
490	0	0	4
491	0	0	4
492	0	0	18
493	0	0	9
494	0	3	18
495	0	0	4
496	0	0	4
497	0	0	4
498	0	0	4
499	0	0	4
500	0	0	13
501	0	0	4
502	0	0	4
503	0	0	4
504	0	0	4
505	0	0	4
506	0	0	4
